# COMPONENTS OF A HEALTHFUL DIET ARE ASSOCIATED WITH WAIST CIRCUMFERENCE AMONG PRE- AND POSTMENOPAUSAL WOMEN

**DOI:** 10.1093/geroni/igz038.214

**Published:** 2019-11-08

**Authors:** Lisa M Troy, Sarah Witkowski

**Affiliations:** 1 University of Massachusetts Amherst, Amherst, Massachusetts, United States; 2 Smith College, Northampton, Massachusetts, United States

## Abstract

After menopause, women are at increased risk of diabetes and cardiovascular disease. A contributing factor to increased risk may be weight gain, especially visceral adiposity. Diet plays a role in maintaining weight at all ages but less is known about the specific contributions of a healthful dietary pattern after menopause. Therefore, we evaluated associations between diet and WC as a measure of visceral adiposity. We compared 869 pre- (aged 35-45 years) and 353 post-menopausal (aged 40-65 years) women from NHANES III (1988-94). Women were pre-menopausal if they self-reported menses in the past 2 months and postmenopausal if they reported no menses in past 12 months and were aged > 40 years. Compared to premenopausal women, postmenopausal women consumed fewer Calories (-200 kcal/d) and had a higher mean waist circumference (+4.43 cm, p=0.007), after adjusting for age, race-ethnicity, height, physical activity, and smoking. Higher intakes of dark green vegetables (p=0.03) and lower intakes of potatoes (p=0.03), refined grains (p=0.001), and meats (p=0.04) were associated with lower WC for all women. Higher intakes of nuts and seeds and fish high in Omega-3 fatty acids were associated with smaller WC while higher intakes of poultry and dairy products were associated with higher WC in post- but not pre-menopausal women. Our findings generally support a diet high in nuts and seeds, dark green vegetables, and fish, and low in potatoes, refined grains, and meats. After menopause it may be important to incorporate fatty fish, nuts and seeds into the diet for lower visceral adiposity.

